# Modeling cell-in-cell structure into its biological significance

**DOI:** 10.1038/cddis.2013.147

**Published:** 2013-05-16

**Authors:** M-f He, S Wang, Y Wang, X-n Wang

**Affiliations:** 1 The Institute of Life Sciences, Chinese PLA General Hospital and SCUT, the State Key Laboratory of Kidney Disease,, Beijing 100853, China; 2 Shanghai Institute of Immunology, Institute of Medical Sciences, Shanghai Jiaotong University, School of Medicine, Shanghai 200025, China

**Keywords:** cell-in-cell, cannibalism, entosis, emperitosis, biological significance

## Abstract

Although cell-in-cell structure was noted 100 years ago, the molecular mechanisms of ‘entering' and the destination of cell-in-cell remain largely unclear. It takes place among the same type of cells (homotypic cell-in-cell) or different types of cells (heterotypic cell-in-cell). Cell-in-cell formation affects both effector cells and their host cells in multiple aspects, while cell-in-cell death is under more intensive investigation. Given that cell-in-cell has an important role in maintaining homeostasis, aberrant cell-in-cell process contributes to the etiopathology in humans. Indeed, cell-in-cell is observed in many pathological processes of human diseases. In this review, we intend to discuss the biological models of cell-in-cell structures under physiological and pathological status.

## Facts

1. Cell-in-cell phenomenon is a common form following cell–cell contact, which is not only observed between tumor cells during tumor cell proliferation and metastasis but also tumor-immune cells suggesting inflammatory responses.
2. Cell-in-cell structures occur under certain physiological circumstance like T-cell development in thymus that thymocyte nurse cells internalize immature thymocytes to nurture and educate them into mature T cells.
3. Cannibalism, entosis and emperitosis (killer cell-involved apoptotic cell-in-cell death) are three types of cell-in-cell death processes undergoing distinct mechanisms.
4. Two models for the outcomes of cell-in-cell are suggested, in which heterotypic cell-in-cell formation is defined as an ‘in-cell' danger signal. That may facilitate the ‘in-cell' self and non-self recognition and trigger the most efficient self-protective mechanisms according to the type of internalized cells.

## Open Questions

1. What are the triggering factors to initiate cell-in-cell structure formation under inflammation or tumorigenesis?
2. What are the exact mechanisms of the vacuolar structure formation during homotypic and heterotypic cell-in-cell progresses?
3. What is the molecular basis of ‘in-cell' signals after different types of cell-in-cell structure formation for undergoing either entosis or emperitosis?
4. Is the interruption of cell-in-cell structure formation a promising strategy to retard the progression of cancer?

Cell-in-cell structure formation describes a process by which one or more cells, that is, effector cells, penetrate into the cytoplasm of another cell, that is, host or target cell, and cause cell structure and biological alteration (‘effector cell' denotes the cell penetrating another cell and ‘host cell or target cell' denotes the cell that has been penetrated). It can be found with low species entering low species or low species entering high species. Zooxanthellae in coral polyps is an example of one type of microorganism inside another type of microorganism to achieve commensalism. Plasmodium completes their life cycles by utilizing different host cells as the carrier. In mammalian cells, fertilization is perhaps the most well-known phenomenon when one mammalian cell enters another mammalian cell. Although the description of this phenomenon under pathological status can be traced back to more than 100 years ago, there have been few reports about the biological significance of cell-in-cell phenomenon.^1^ Cell-in-cell can take place among the same type of cells (homotypic cell-in-cell) or different types of cells (heterotypic cell-in-cell). The types of effector and target cells vary extensively from terminally differentiated cells to stem cells, from immune cells to non-immune cells, and from normal tissue cells to abnormal cells.^2, 3^ Apart from autologous internalization of tumor cells, immune cells are the most frequent effector cells, whose entering into other cells is termed emperipolesis early.^4^ In recent years, there have been more thorough researches into the occurrence and fate of cell-in-cell structures formed among homotypic and heterotypic cells as well as their mechanisms and potential biological significance. Several intracellular death processes, including cannibalism, entosis and killer cell-mediated intracellular apoptosis and their mechanisms have been studied in more detail, and their potential biological significance has attracted more attention.^3, 5, 6^ The biological effects on characteristic alteration of target cells during cell-in-cell processes are emerging.^7, 8^

Study of cell-in-cell has also been evolved from biological significance to its roles on the development of diseases.^9, 10, 11^ Obviously, the roles cell-in-cell plays on the occurrence and development of diseases, which has been largely ignored previously, will be the focus of future researches with more attention. In this review, based on new comprehensive research progresses in this field, we aim to look further into the working models of cell-in-cell with the hope to enrich the knowledge of biological significance as well as its putative roles in the development of disease.

## Cell-in-Cell Structure: a Place for Cellular ‘Face Transplant Surgery'?

Thymic nurse cells (TNCs) represent the most typical physiological case of living cells internalizing other living cells and of internalized cells being released from the host cell's cytoplasm with altered biological characteristics. TNCs were first discovered in mouse thymuses as reported by Wekerle and Ketelson in 1980.^12, 13^ They found that the cytoplasmic vacuoles of thymic epithelial cells expressing keratin contained many completely internalized thymic cells, ranging from approximately 7 to 50 cells. The thymocytes invading rather than being phagocytosized into thymic epithelial cells showed significant mitotic activity.^12^ TNCs are special thymic epithelial cells expressing a specific cell marker pH91.^14^ Major histocompatibility complex molecules are also expressed on cell surface as well as on the surface of vacuoles containing internalized thymocytes in cytoplasm.^15^ TNCs only internalize immature *αβ*TCR^low^CD4^+^CD8^+^ thymocytes. Through a ‘face transplant surgery' driven by major histocompatibility complex molecules inside the TNCs, these internalized immature T cells differentiate into *αβ*TCR^high^CD4^+^CD8^+^CD69^+^ cells that possess mature T-cell markers followed by escaping from the TNCs.^16^ TNCs have not only a positive-selection function that promotes TCR remodeling but also a negative-selection role, which selects anergy thymocytes to be eliminated early in the intracellular death processes. The role of TNCs in T-cell development is still under debate as the dominant theory holds that negative selection occurs in thymic medulla rather than in the cortex.^16, 17, 18^ Recently, the work of Hendrix *et al.*^19^ has shown that TNCs simultaneously express K8 cytokeratin, K5 cytokeratin, P63 as well as AIRE and TRA, which facilitate the negative selection through expression of autoantigens by TNCs. Furthermore, they can be located in the junction between thymic cortex and medulla. This reveals that there might exist different TNC subsets that perform different functions. Interestingly, Samms *et al.*^20^ found that macrophages from peripheral blood also enter into TNCs and participate in positive and negative selection of internalized immature T cells.

The fact that cells gain new biological characteristics in TNCs *via* cell-in-cell process reminds us of certain events observed in lower species.^21, 22, 23^ For example, the life cycle of plasmodium involves entering and maturing in human hepatocytes and erythrocytes, resulting in malaria.^21, 22^ In these events, host or target cells are usually ruptured after releasing the internalized cells. By contrast, the escape of the effector cells in mammals does not result in the destruction of target cells.^24^ This is similar to a symbiosis state established by cell-in-cell structures seen in lower species, such as zooxanthellae stationing inside cells of coral polyps.^25^

Similar observation is reported that liver epithelial cells act as nursing cells to promote the maturation of erythrocytes^26^ or eliminate auto-reactive immune cells through negative selection to maintain homeostasis.^27^ Benseler *et al.*^27^ recently found that most naive autoreactive T cells, being adoptively transferred into recipient animals, concentrated in the liver and were internalized into epithelial cells. These autoreative T cells underwent cell-in-cell death through lysosomal degradation instead of caspase pathway. When they were blocked from internalization, autoreactive T cells increased significantly in peripheral blood and liver tissue, which, in turn, caused autoimmune damage to the recipients. These results further suggest that cell-in-cell formation is more prevalent than expected during cell development, differentiation and homeostasis. Despite having been searched extensively during B-cell development,^28^ no ‘B nurse cell' phenomena have been found until now. However, certain follicular dendritic cells might use the internalization of other cells to perform nursing B-cell development.^29^

A very interesting event observed frequently in the previous *in vitro* cell-in-cell research but difficult to explain in detail is the escape of the effector cells from target cells, even those undergoing mitosis inside target cells.^3, 6^ Whether or not the effector cells that escape from target cells change their biological characteristics is still unknown.

## Cell-in-Cell Structure: a Battlefield or a Slaughterhouse?

It is revealed earlier that the effector cells entering target cells remain alive and active. Early reports showed that some of immune cells, after internalization, could attack tumor cells by directly inserting into the nucleus of target cells.^30, 31, 32^ However, the main fate of most internalized effector cells has been shown as undergoing cell-in-cell death. There are three types of cell-in-cell death resulting from cell-in-cell structures, including cannibalism, entosis (non-apoptotic cell-in-cell death) and emperitosis (killer cell-mediated apoptotic cell-in-cell death).^3, 5, 6^

The most systematically investigated cell-in-cell death process is cannibalism in cancer.^5, 33, 34, 35, 36^ Fais and Fauvarque^33^ demonstrate that tumor cells under starvation conditions can ‘eat' neighborhood tumor cells and even immune cells. By eating these cells, they increase their proliferative capacity and promote the malignancy. This coincides with the concept raised recently that tumors are a new type of cell species evolved *in vivo*.^37^ Tumor cells may ‘eat' other cells in order to increase their autonomy and gain stronger invasiveness and potential for metastasis, thus resulting in a worse clinical prognosis. Cannibalistic cells use caveolin-1, ezrin and actin to efficiently consume the cells in contact with its outer membrane. A cannibalistic vacuole is formed and likely to fuse with lysosome to form caveosomes rich in cathepsin-B. ATPase-mediated acidification in the caveosomes maintains cathepsin-B in activated status to mediate the degradation of consumed cells.^5, 36^ Recent investigation shows that a nine transmembrane segment (TM9) of TM9SF4 in phagocytosis is also involved in cannibalism of melanoma cells and phagocytes.^33^ Further studies on cannibalism of pancreatic ductal adenocarcinoma indicate that the engulfed cells undergo nuclear fractionation through caspase-3 dependent apoptotic cell death.^34^ Interestingly, engulfing cells can survive under starvation conditions, whereas cells that engulf plastic beads cannot. This observation further demonstrates that cannibalism is one of the survival mechanisms of malignant cells under starvation.^35, 36^

Another new cell-in-cell death pathway attracting more attention recently has been termed as entosis. Similar to cannibalism, entosis occurs between two homotypic cells and involves tumor cells ‘eating' tumor cells. Although entotic cell-in-cell structure is similar to that of cannibalism or phagocytosis, homotypic entosis is a process in which a living tumor cell invades intactly into a neighboring cell of the same type. Under starvation condition, effector cells perform entosis, which is similar to autophagy for survival. During entosis, the effector cells are enveloped in the vacuole of target cells, which promote Light Chain 3 (LC3) recruitment from target cells onto the entotic vacuole membranes. The translocation of LC3 depends on autophagy lipidation machinery such as autophagy protein 5 (Atg5), Atg7 and the lipid kinase VPS34 (vacuolar protein sorting 34) rather than autophagosomes. After entotic vacuoles fuse with lysosomes of target cells, effector cells are deleted by target cells. Therefore, entosis is a unique type of cell-in-cell, non-autophagosome-dependent lysosomal death pathway.^38^

We observed 30 years ago that mouse spleen natural killer (NK) cells killed cancer cells after internalization. However, >70% of internalized NK cells were self-degraded inside tumor cells and exhibited typical apoptotic morphology. This suggests that tumor cells may be able to ‘strike back' to kill those immune cells inside.^39^ Based on our recent work by using NK92 cell line and human tumor cells for study, we found that cell-in-cell death process of NK92 inside tumor cells was a typical caspase-3-dependent apoptosis differing from either entosis or cannibalism.^6, 40^ Takeuchi *et al.*^41^ also reported the apoptotic death of a cytotoxic regulatory T-cell line inside tumor cells. These observations suggest a new type of cell-in-cell death pathway occurring through interaction between heterotypic cells, especially between immune cells and tumor cells. However, by analyzing a series of tumor and immune cell lines, we found that the type of cell-in-cell death mainly depended on the properties of the effector cells.^42, 43^ Only those with cytotoxic property such as NK cells or cytotoxic T cells underwent cell-in-cell death in a caspase-3-dependent pathway. Those without cytotoxicity, like B cells or monocytes underwent typical entosis after they were internalized into tumor cells.

Further study illustrates that activated granzyme B (GzmB) existing in intracellular cytotoxic cells cannot directly get into the cytosol of target cells due to vacuole formation. However, it is rationale that cytotoxic effector cells might rapidly release pre-existing GzmB in the endosome and leak some into the cytosol of target cells. This results in a re-picking-up of the active GzmB back into the internalized cells and induces the cell-in-cell apoptosis afterwards,^42, 43^ just as the suicide of killer cell inside tumor cells. These results further support our observations made 30 years ago.^39^ In order to distinguish between the aforementioned two types of cell-in-cell death, we define this type of caspase-3-dependent apoptotic cell-in-cell death as emperitosis (taken from emperipolesis and apoptosis).

Concerning the biological significance, entosis may have two contradictory biological effects. On the one hand, it ‘inhibits' tumor metastasis by discarding internalizing cells that detach from the extracellular matrix.^3^ On the other hand, the effector cell induces a certain percentage of multinucleated or aneuploid target cells by blocking the cytokinesis of target cells. Chromosome instability (CIN) is also observed leading to the further malignancy of target cells through cell fusion.^7, 8^ However, if taken target tumor cell with internalized effector cell as an entity, the killing of internalized cells through entotic effects by target cells should be considered as a homeostasis mechanism to maintain internal stability.

In emperitosis, the death of cytotocxic killer cells inside tumor cells is just like soldiers killed by the bounced-back bullet. It can also be considered as a slaughtering action of tumor cells to ‘fight back', a strategy of tumor cells escaping from immune surveillance. According to this opinion, the tumor/immune cell-in-cell phenomenon may be taken as a tumor prognosis marker. The molecules involved in this process might serve as new drug targets with therapeutic effects.^5, 35^ *In vivo* disease models could be used to elucidate the underlying significance of the process in order to reflect the pathogenic roles that cell-in-cell has in the development of diseases.

In summary (Table 1), four types of cell-in-cell death (phagocytosis, cannibalism, entosis and emperitosis) exhibit both shared and unique characteristics. What is common in that cell-in-cell death of either immune or tumor cells within tumor cells is suggested to be the manifestation of tumor cells' autonomy. By ‘eating' these effector cells, tumor cells get more nutrients or chromosomal contents from them and become more competitive in proliferation and invasiveness.

## Cell-in-Cell Structure Formation: an *in situ* Activity or a Holistic Regulatory Reaction, especially in the Development of Diseases?

Cell-in-cell phenomena have gained more attention over the recent years after being ignored for almost a century.^9, 11, 40, 44, 45^ Their biological mechanisms^3, 6, 34, 35^ and pathogenic roles are starting to emerge.^7, 10, 27^ Although some investigators questioned the cell-in-cell processes as an *in vitro* phenomenon, almost all observations of cell-in-cell structures were reported from clinical biopsy specimens.^47^ In some particular cases, cell-in-cell structures have become a specific characteristic of the diseases, such as Rosai-Dorfman disease, chronic myeloproliferative diseases and some hematological diseases.^46, 48, 49, 50^

The roles of cell-in-cell structure formation in tumorigenesis are still under debating. Schools of thought are prone to support that cannibalism is beneficial for tumor promotion and associated with clinical deterioration in cancer cases.^5^ When examining clinical urine specimens, Gupta *et al.*^51^ discovered that cannibalistic activity, degree of malignancy and metastatic potential of malignant cells were closely related. The higher the malignancy factor, the more common cannibalism there was. This suggests that cannibalism may be used as one of the indexes of tumor malignancy in morphological diagnosis and exerts its therapeutic potential by interfering this process.^5, 52, 53, 54^ Like cannibalism, immune cells undergoing emperitosis are also victims of tumor cells with an obvious tendency toward promoting tumorigenesis,^6, 35^ which raises new interests for researchers.

Study of cell-in-cell structure formation in autoimmune disease by Benseler *et al.*^27^ has brought a new thread for explaining the roles of cell-in-cell structure formation during pathogenesis. Their results also imply that cell-in-cell structure formation is an inherited as well as evolutionarily conserved manner of cell–cell interaction in organisms and can be used as a homeostasis mechanism at the holistic level. From this point of view, many questions about cell-in-cell phenomenon need to be answered. For example, during the process of liver epithelial cells eliminating autoreactive T cells, how can organisms respond properly to such danger signals and balance the pros and cons? What kinds of mechanisms are used to mediate these T cells penetrating into epithelial cells and eliminate the autoreactive T cells? Association study on cell-in-cell structure formation to the pathogenesis of diseases may lead to a new research wave focusing on exploration of these phenomena.

**Figure 1:**
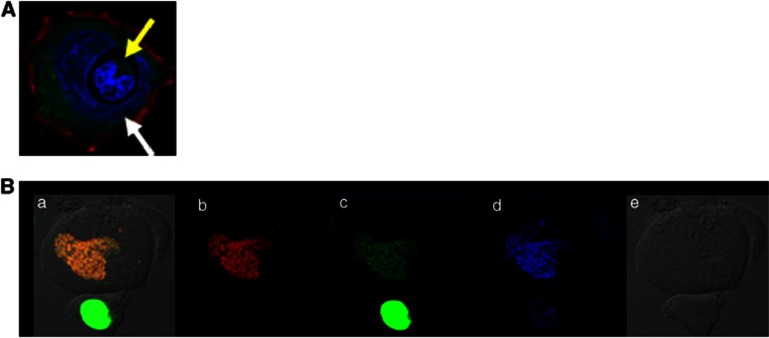
Nucleus penetration and nucleus fusion after cell-in-cell interaction. (**A**) Nucleus penetration One lymphocyte (yellow arrow) penetrates directly into the nucleus of a host cell (white arrow) to form a typical heterotypic cell-in-cell structure. Nucleus is displayed in purple with DAPI (4,6-diamidino-2-phenylindole) staining. (**B**) Nucleus fusion One PLC/PRF/5 cell line expressing H2B-EGFP (green, b) is co-cultured with one PLC/PRF/5 cell line expressing H2B-RFP (red, c) for 4 h. DAPI staining is preformed (blue, d) before cell-in-cell structure is observed under differential interference contrast (DIC) microscopy (e). Yellow nucleus under DIC image is shown after 4 h (a) probably due to cell fusion of two cells. (Unpublished data)

**Figure 2:**
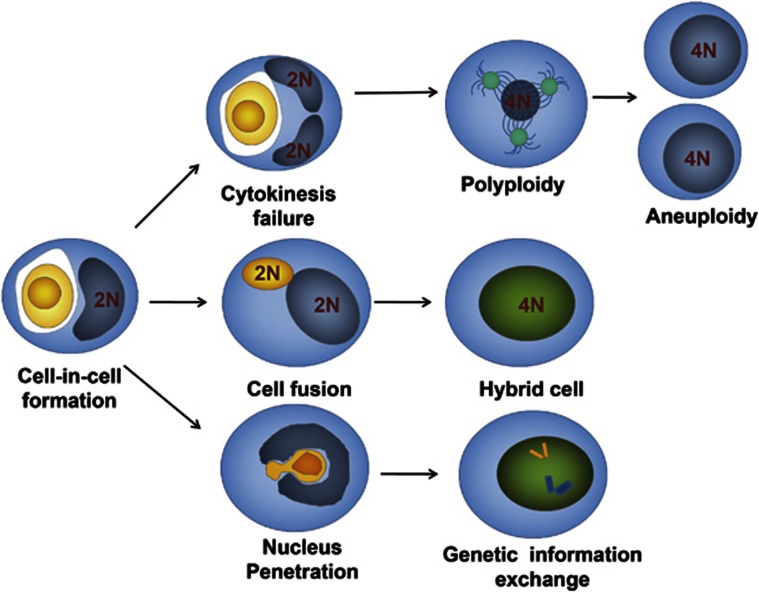
Pathways that cause CIN of target cells through cell-in-cell interaction. Aneuploidy of target cells results from failure of target cell cytokinesis during homotypic or heterotypic cell-in-cell structure formation (top channel). Cell fusion between target cells and internalized cells occurs through cell-in-cell interaction (middle channel). Direct exchanges of genetic material occur in the nucleus between effector cells and target cell (low channel)

The biological outcomes derived from cell-in-cell interaction *in situ* may result in a holistic response, as in the case of autoreactive T-cell elimination through cell-in-cell death mentioned above. Studies from entosis indicate that by retarding the mitosis of target cells, a certain percentage of multinucleated or aneuploid cells in target cells are generated owing to the internalized cells. A straight-forward biological consequence on target cells is the change in their CIN.^6, 7, 8^ We also observed the multinucleated or aneuploid target cells produced by heterotypic immune-tumor cell-in-cell, even normal tissue cells, which is similar to those in homotypic tumor-tumor cell-in-cell structures. The chromosomal components from the effector cells were easily detectable in target cells after heterotypic cell–cell interaction. Internalized cells cause CIN of target cells probably by exchanging chromosomal components through penetrating directly into the nucleus of target cells or fusing with them^30, 31, 32^ (Figure 1). More strikingly, we found that cell-in-cell phenomenon was commonly observed in inflammation (such as mouse hepatitis and graft-verse-host disease models), tumor and other clinical diseases.^43^ According to the recent prevailing theory, inflammation is an accelerator of tumorigenesis partially due to the release of the inflammatory substances to induce CIN.^55, 56^ The high tendency to form cell-in-cell structure in inflammation might represent one novel mechanism to form aneuploid cells in local, which might promote transformation of normal cells. With increased frequency of CIN induced in inflammation by cell-in-cell structure formation, we speculate that cell-in-cell structure formation might become a ‘fast track' from inflammation toward cancer transformation.^43^ New *in vivo* cell-labeling techniques, high-resolution fluorescence imaging, nano-scale imaging techniques, tracking technologies and animal models utilizing chemically induced inflammation toward cancer^57, 58, 59^ will be used to reveal the exact roles played by cell-in-cell structure formation during tumorigenesis. CIN caused by cell-in-cell structure formation is depicted in Figure 2.

**Figure 3:**
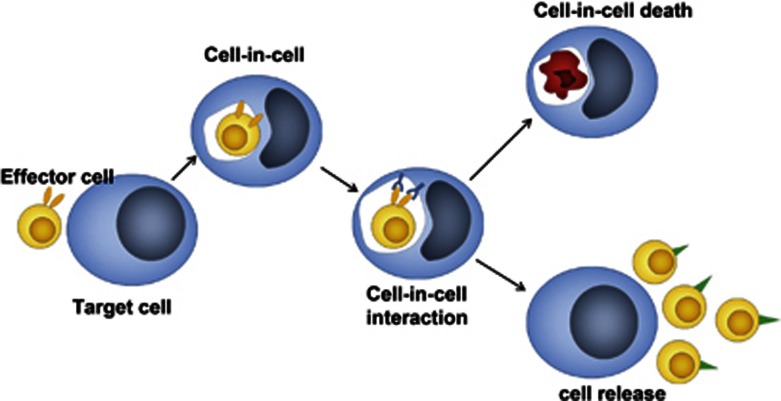
A cell-in-cell selection model. We propose this model according to the reports that immature thymocytes penetrate into thymocyte nursing cells (TNCs) for nutrition and maturation. Accordingly, effector cells (like immature thymocytes) enter into target cells (TNCs) to form cell-in-cell structures. After cell-in-cell interaction, some internalized effector cells can be released with new properties (low panel). The others are trapped in the vacuole and energized or eliminated inside target cells (upper panel)

We thus propose two models about the biological effects of cell-in-cell processes in mammalian cells. The first hypothetic effect is a cell-in-cell selection model (Figure 3). In this model, some cells, such as T-cell precursors, enter into certain target cells and change their properties inside to gain new biological traits. Cells released from target cells carry out new biological functions. TNCs are an example of this model. Some internalized effector cells are eliminated through an entotic mechanism by target cells to achieve homeostasis, an example being the fate of autoreactive naive T cells in a normal animal host.^27^

**Figure 4:**
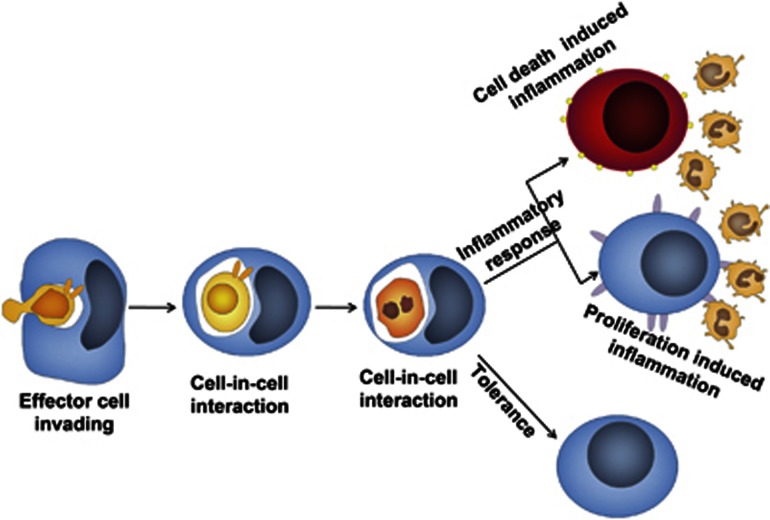
A cell-in-cell stress model. When one effector cell (immune cells) enters into one target cell (tissue or tumor cell), a special niche will be established around them to induce either inflammation or immune tolerance. Cell death or cell proliferation inside target cells contribute to inflammatory response while the elimination of effector cells inside target cells leads to immune tolerance

The second hypothetic effect is a cell-in-cell stress model (Figure 4). In this model, effector cells invade into specific target cells and cause the death of either target or effector cells through intracellular interactions. However, the key point is that through intracellular interactions a series of changes happen to target cells, including gene expression, membrane molecules and cytokine secretion patterns and so on. These changes turn target cells into a new biological entity and cause cell plasticity in peripheral microenvironment like inflammation. In the case of autoreactive naive T cells mentioned above, local and overall inflammation might be suppressed by elimination of infiltrating T cells inside host liver epithelial cells, which would be an example of the first model.^27^ However, we also assume that the suppressive effect may result from immune tolerance induced by liver epithelial cells interacting inside with internalized autoreactive T cells and the acquisition of new biological characteristics, thereby inducing an immune tolerance-dominant microenvironment. If this hypothesis is true, we could speculate that either severe fulminate hepatitis or an asymptomatic virus carrier state after hepatitis virus infection would be determined by liver epithelial cells that undergo different cell-in-cell biological behaviors with liver–immune cell interaction. We have already observed that the occurrence of cell-in-cell structures was apparent in hepatitis of mouse model and human. Moreover, lineage transition of immune effector cells are observed in the different stages of the disease, with NK cells in the early stage and granulocyte leukocytes in the late stage.^43^ The differences in cell types may lead to different biological effects after heterotypic cell-in-cell interactions, all worthy of further investigation.

## Conclusion and Perspective

Cell-in-cell phenomenon is a common form following cell–cell contact, which has been long overlooked. Cell-in-cell structures are not only frequently observed between tumor cells during tumor cell proliferation and metastasis but also tumor-immune cells in inflammatory responses. The unexpected high frequency of cell-in-cell occurring either *in vitro* or *in vivo* suggests that this process represents an evolutionally conserved cell–cell interaction, which has critical roles in development and homeostasis. The biological properties of effector or target cells after cell-in-cell interaction as well as its involvement in pathogenesis need to be further investigated.

In addition, lysosomal degradation-involved entosis and apoptosis-involved emperitosis represent two cell-in-cell death pathways determined by effector cells when target cells sense different in-cell signals triggered by cell-in-cell formation. However, it is still difficult to explain why entosis undergoes lysosomal degradation while emperitosis undergoes apoptosis (either caspase or cytochrome C triggered). It is possible that these two processes endow with different mission for internalized cells: for entosis, target cells degrade homogenous cells for self nutrition and proliferation, whereas heterotypic cell-in-cell structure is more likely to be an ‘in-cell danger signal' whose destination is to completely eliminate effector cells by apoptosis. To some extent, emperitosis might be the alternative form of entosis with different aims of cellular biological behavior. To elucidate the exact biological significance will facilitate our understanding of how cell-in-cell initiates the ‘in-cell' self and non-self recognition for the most efficient self-protection according to the type of internalized cells.

Finally, as vacuolar structure formation is demonstrated to be the key checkpoint in cell-in-cell structure formation, how homotypic and heterotypic cell-in-cell structures form and provide different in-cell signals to undergo either entosis or emperitosis might become future research focus. To elucidate the detailed mechanisms will probably provide new strategies for target screening in multiple diseases. In addition, studies on pathogenic mechanisms of cell-in-cell formation during pathogenesis will provide new targets for drug development and treatment regimens.

## Figures and Tables

**Table 1:** Characteristic summarization of cell-in-cell

	*Phagocytosis*	*Cell-in-cell*		
		*Cannibalism*	*Entosis*	*Emperitosis*
Types of effector cells	Apoptotic cells	Dead or live cells	Live cells	Live cells
Types of target cells	Phagocytes	Tumor cells	Tumor cells	Tumor cells or normal cells
Fates of effector cells	Degradation	Cell death	Cell death or mitosis or release	Cell death or mitosis or release
Triggering factors	Phosphatidylserine (PS) on apoptoic cells	Starvation	unknown	unknown
Engulfment of effector cells	Cytoskeletal rearrangements	Adherens junctions	Adherens junctions; Rho-ROCK signaling pathway; myosin-based contractile force (ref 60)	Adherens junction Rho-ROCK-Actin/myosin pathway
Molecules participating in the processes	PS, CD14, CD68, vitronectin receptor (VNR)	Caveolin-1, actin, ezrin, cathepsin B, a nine transmembrane segment (TM9SF4), vimentin	LC3, Atg5, Atg7, Rho, ROCK, Vps34, cadherin	LFA-1, ICAM-1, CD62, Ezrin, ICAM-2, E-cadherin
Cell death pathway	Lysosome-mediated degradation	Lytic enzymes mediation	Lysosome-mediated caspase-3 independent cell death	Apoptosis
Biological function	Removal of pathogens and cell debris to maintain the internal homeostasis; nourishment of target cells	Nourishment of target cells	suppression of transformed growth; induction of aneuploidy	Tumor escape; acquisition of nutrients; nursing of immature T cells

## Abbreviations

TNC: thymic nurse cell
VPS34: Vacuolar Protein Sorting 34
ATG5: Autophagy Protein 5
ATG7: Autophagy Protein 7
LC3: Microtubule-associated Protein1A/B Light Chain3
ECM: Extracellular Matrix
NK: natural killer
CIN: chromosome instability
